# Functional versus effector-specific organization of the human posterior parietal cortex: revisited

**DOI:** 10.1152/jn.00312.2014

**Published:** 2016-07-27

**Authors:** Tobias Heed, Frank T. M. Leone, Ivan Toni, W. Pieter Medendorp

**Affiliations:** ^1^Donders Institute for Brain, Cognition and Behaviour, Radboud University Nijmegen, Nijmegen, The Netherlands;; ^2^Biological Psychology and Neuropsychology, University of Hamburg, Hamburg, Germany; and; ^3^Biological Psychology and Cognitive Neuroscience, Bielefeld University, Bielefeld, Germany

**Keywords:** cortical organization, foot, parietal cortex, pointing, sensorimotor processing

## Abstract

It has been proposed that the posterior parietal cortex (PPC) is characterized by an effector-specific organization. However, strikingly similar functional MRI (fMRI) activation patterns have been found in the PPC for hand and foot movements. Because the fMRI signal is related to average neuronal activity, similar activation levels may result either from effector-unspecific neurons or from intermingled subsets of effector-specific neurons within a voxel. We distinguished between these possibilities using fMRI repetition suppression (RS). Participants made delayed, goal-directed eye, hand, and foot movements to visual targets. In each trial, the instructed effector was identical or different to that of the previous trial. RS effects indicated an attenuation of the fMRI signal in repeat trials. The caudal PPC was active during the delay but did not show RS, suggesting that its planning activity was effector independent. Hand and foot-specific RS effects were evident in the anterior superior parietal lobule (SPL), extending to the premotor cortex, with limb overlap in the anterior SPL. Connectivity analysis suggested information flow between the caudal PPC to limb-specific anterior SPL regions and between the limb-unspecific anterior SPL toward limb-specific motor regions. These results underline that both function and effector specificity should be integrated into a concept of PPC action representation not only on a regional but also on a fine-grained, subvoxel level.

## Abstract (precis)

*In the present study, we show that regions in posterior parietal regions process information independent of the currently used effector (hand, foot, or eye) during goal-directed actions. Functional MRI repetition suppression analysis suggests that generality across effectors holds also on the neuronal level and not just at the level of entire regions. More anterior parietal regions process information only for a specific effector or a subset of effectors*.

## NEW & NOTEWORTHY

*In the present study, we show that regions in posterior parietal regions process information independent of the currently used effector (hand, foot, or eye) during goal-directed actions. Functional MRI repetition suppression analysis suggests that generality across effectors holds also on the neuronal level and not just at the level of entire regions. More anterior parietal regions process information only for a specific effector or a subset of effectors*.

the posterior parietal cortex (PPC) is known as a key structure in sensorimotor integration (Andersen and Cui 2009; Blangero et al. 2009; Medendorp et al. 2011). The region is divided into several subregions with specific short-range connections as well as extensive connections with frontal regions (Rizzolatti et al. 1998). However, its overarching organizational structure is under debate.

From a motor perspective, there is abundant evidence that the PPC distinguishes the processing for eye and hand movements, especially in the monkey (Andersen and Cui 2009; Chang et al. 2008; Premereur et al. 2015), although this division appears gradual rather than absolute, especially in humans (Caminiti et al. 2010; Filimon et al. 2009; Gallivan et al. 2011; Heed et al. 2011; Hinkley et al. 2009; Leoné et al. 2014; Tosoni et al. 2008). These observations have traditionally been interpreted in terms of an effector-specific organization of the PPC. However, recent imaging studies have suggested that the organization in the PPC may instead be guided by other functional aspects.

For example, observation of actions performed by another individual activated the PPC in relation to the type of action rather than the effector with which the action was performed (Abdollahi et al. 2013; Jastorff et al. 2010). Similar results were obtained for motor imagery of own hand and foot actions (Lorey et al. 2014). Furthermore, executing signing movements with the hand and foot has revealed overlapping activation within the intraparietal sulcus (IPS) (Rijntjes et al. 1999). We have recently reported that the planning of goal-directed hand and foot movements evoked markedly similar PPC activation, whereas the activation evoked by the planning of eye movements (saccades) was different from limb-related activation (Heed et al. 2011). In line with these findings, flexion of the wrist and ankle according to a fluctuating visual cue led to overlapping activation in several PPC regions (Cunningham et al. 2013). All these studies suggest that effector specificity is not a defining processing feature of the PPC and that, instead, the PPC may be organized according to functional criteria rather than in an effector-specific manner. In such a scheme, differences in the processing of saccade planning would be due to the different functional role played by the eyes compared with other effectors.

However, regular fuctional MRI (fMRI) contrast analysis usually reveals regional activations in the order of several millimeters to centimeters and does not further assess the information contained within these large activation patterns. To remedy this limitation, we recently investigated the planning of eye, hand, and foot motor planning with a combined activation and multivoxel pattern analysis (MVPA) (Leoné et al. 2014). Activation was observed for several effectors in many PPC regions, and their voxel patterns were informative about effector selectivity. Thus, MVPA revealed effector-specific coding where traditional activation analysis did not.

However, it remains unknown how these distinctions are implemented at the neuronal level. While MVPA examines the clustering of effector selectivity across voxels in a region, it does not distinguish the fine-grained neural organization within the voxels. An approach that could further interrogate neural representations is fMRI repetition suppression (RS), also known as fMRI adaptation. It is based on the finding that neural and hemodynamic responses are reduced when the feature to which a region responds is repeatedly presented (Desimone 1996; Grill-Spector and Malach 2001; Sawamura et al. 2006). Although the specific relationship of RS effects in fMRI with single neuron responses is not yet clear (Krekelberg et al. 2006) and may differ across different brain regions, single cell recordings in monkeys have suggested that the presence of RS effects in fMRI also indicates the presence of RS effects at a neuronal level (Grill-Spector 2006; Sawamura et al. 2006). Thus, the underlying logic is that fMRI RS will be observed if two consecutive stimuli drive, at least in part, the same neurons because they share a characteristic relevant to the region under investigation. fMRI RS therefore allows inference about neuronal coding within an fMRI voxel.

Here, we used fMRI RS to distinguish two possible types of regional organization. Neurons in the areas that are similarly activated by the hand and foot may have responded equally well to both limbs. Such behavior would be expected if these neurons process stimulus (rather than effector) characteristics or if they code movement-related parameters in a reference frame common to all effectors (Batista et al. 1999; Buneo and Andersen 2006; Hagler Jr. et al. 2007; Medendorp et al. 2005). Alternatively, such regions may contain two separable pools of specialized neurons, one for the hand and one for the foot, that are spatially arranged in an intermingled manner. Thus, if a region contains separate neuronal pools for different limbs, then an RS effect should be obtained if a given limb is used repeatedly but not if it is used following on the other limb. According to similar logic, a region whose neurons favor the limbs over the eyes should show an RS effect only between limbs and eyes but not between the two limbs.

## METHODS

### Ethics

The present study was conducted at the Donders Institute in Nijmegen, The Netherlands. It was conducted according to the guidelines of the Declaration of Helsinki in its latest version and was approved by the ethical committee of the German Research Foundation as well as by the local ethics committee (CMO Committee on Research Involving Human Subjects, region Arnhem-Nijmegen, The Netherlands).

### Participants

Twenty-three participants took part in the experiment. Seven participants were not included in the analyses because too many trials were eliminated during preprocessing (see below). The remaining 16 participants (9 women and 7 men) were aged 19–33 yr (mean: 23.5 yr). All were righthanded and rightfooted by self-report, had normal or corrected-to-normal vision, and reported to be free of any neurological disorders. None of the participants had participated in our previous study (Heed et al. 2011).

### Eye Tracking and Hand and Foot Movement Recording

The study's setup was similar to that of our previous study and is described in detail there (Heed et al. 2011). In brief, eye position was recorded at a sampling rate of 50 Hz using a long-range infrared video-based eye tracker (SensoMotoric Instruments). Eye fixations and saccade onsets were identified offline. Saccade reaction time was defined as the time between the movement cue and saccade onset. To measure hand and foot movements, infrared LEDs were attached to the right hand and foot. These LEDs were recorded continuously by a camera during the experiment, and limb movements and their reaction times were identified offline. Limb movement amplitude was assessed as the distance in pixels between pointing start and end positions. This measure allows relative comparisons between movement amplitudes for different target eccentricities.

### MRI Recording

#### fMRI measurement.

Functional images were acquired on a Siemens 3 tesla MRI system (Tim TRIO, Siemens) using a 32-channel phased array head coil. Using a multiecho sequence, we obtained 26 axial slices with a thickness of 3 mm, a gap of 0.5 mm, an in-plane pixel size of 3 × 3 mm at a repetition time (TR) of 2,010 ms, echo times (TE) for the five echoes of 9.4, 21.2, 33, 45, and 57 ms, respectively, a field of view of 192 mm, and a flip angle of 80°. Measurements covered the entire parietal cortex, the motor-related regions of the frontal lobe, and the majority of the occipital lobe.

#### Anatomic MRI measurement.

After functional recordings, we acquired 1 × 1 × 1-mm resolution anatomic images using a T1-weighted MPRAGE sequence with 176 sagittal slices and a field of view of 256 mm at a TR of 2,300 ms, a TE of 3.93 ms, and a flip angle of 8°.

### Experimental Setup and Task Design

Participants lay supine in the MR scanner and were cushioned underneath their right leg. The upper arm and the upper legs were strapped to the scanner bed to reduce potential movement during pointing. The experiment was executed in the dark, with the exception of a beamer display on which instructions and targets were presented approximately above the participant's head. Participants made pointing movements with the extended right index finger, right big toe, and eyes. For finger pointing, the index finger pointing was executed by moving the wrist but not the rest of the arm. For toe pointing, participants moved the ankle. The hand and foot pointed in the horizontal (left-right) direction only, as the degrees of freedom of wrist and ankle limit vertical movement. Participants had to fixate a central dot presented on the beamer display throughout the experiment unless eye movement execution was required. Analogously, the finger and toe had to be pointed toward the fixation dot.

Each trial consisted of a stimulus, a planning phase, and a movement execution phase. At the beginning of a trial, the fixation dot changed color to indicate the effector to be used (red, green, and orange for the hand, foot, or eye), and, at the same time, a light gray target was presented at one of six possible locations (3 to the right and 3 to the left of fixation, all in the same vertical position as the fixation dot). The effector cue and target were shown for 400 ms, after which the fixation dot was shown for a variable time (2–6 s including the cue, square distribution). Participants had to remember the target location and plan the movement. A color change of the fixation dot to purple signaled that the instructed movement to the remembered target location, and a movement back to point towards fixation, had to be executed immediately (2 s including the cue).

The key manipulation of the present study was that the effector to be used in a given trial was either identical to the previous trial (termed “repeat” from hereon) or not (“nonrepeat”). The target stimulus could occur on the same spatial side as in the previous trial but was never presented in the identical location as in the previous trial. We chose this design because the focus of the study was on the representation of different effectors, not on spatial processing. Accordingly, to avoid confounds for the analysis of this feature, the only attribute that was repeated was the effector but never the stimulus location. The trial sequence was balanced using Euler circuits (Brooks 2012) so that it contained every possible combination of consecutive effector and target location (with respect to target side) with equal probability.

The experiment comprised 18 runs of ∼4 min each. Each run started with a 20-s interval in which the participant fixated on the central dot. These intervals served as a baseline in the general linear model (GLM) analysis. The experiment was conducted in two sessions of nine runs each, separated by a short break during which participants left the MR scanner for rest. In total, the experiment comprised 774 trials. Because the first trial of a given run did not have a predecessor, first trials were not analyzed. Of the remaining 756 trials, half were repeats and half were nonrepeats. We acquired this high number of trials for two reasons. First, fMRI RS effects are typically small and, thus, a higher number of trials may be necessary to obtain sufficient statistical power. Second, when participants made an error in a given trial, this trial had to be excluded from analysis, resulting in the exclusion also of the following trial, for which the erroneously executed trial would have been a repeat or nonrepeat predecessor. We therefore excluded a comparably high number of trials from analysis. The criteria for exclusion were breaking of eye fixation (e.g., eye movement in the retention phase or eye movement along with an instructed hand or foot movement), hand and foot movement during the fixation and retention phases, use of the wrong effector in the movement phase, and lack of any movement during the movement phase. Although the task was practiced in advance, these strict criteria led to the exclusion of >40% of trials (including correct trials that followed on error trials) in 7 of our 23 participants. Although a GLM analysis including all 23 participants revealed qualitatively similar results to the GLM including only the remaining 16 participants, we included only those participants of which we could include at least 60% (range: 60–92%) of conducted trials. The main reason for excluding participants were eye movements that accompanied instructed hand and foot movements.

### Analysis of Behavior

Reaction times for eye and limb responses after the movement cue were analyzed with ANOVA. Saccade errors, that is, saccades toward target stimuli during the planning phase and saccades accompanying instructed limb movements, were analyzed with respect to whether they occurred after a saccade trial or after a limb trial to test whether difficulty of suppressing eye movements differs depending on trial history. Error probabilities were analyzed with a generalized linear mixed model (Jaeger 2008), and significance was assessed using likelihood ratio testing.

### fMRI Analysis

A recent report analyzed the data obtained in the present study using a combined MVPA and activation approach (Leoné et al. 2014), collapsing over all trials executed with a given effector. Here, we analyzed fMRI RS effects and, consequently, focused on the effect of trial repetitions.

#### Preprocessing and data analysis.

The five echoes of the functional data were corrected for head motion in SPM8 (Statistical Parametric Mapping, http://www.fil.ion.ucl.ac.ul/spm) and merged using the PAID algorithm (Poser et al. 2006) in Matlab (Mathworks, Natick, MA). The resulting combined functional images were imported into BrainVoyager QX version 2.6 (Brain Innovation, Maastricht, The Netherlands). Here, further preprocessing included slice scan time correction, slow drift correction, alignment to anatomic scans, and spatial transformation into Talairach space. The boundary of the white and gray matter was identified in the anatomic images of each participant, and the cortical sheet of both hemispheres was reconstructed, inflated, and morphed to a sphere (Goebel et al. 2006). The same-side spheres of all participants were then averaged based on their gyral and sulcal patterns (van Atteveldt et al. 2004). Functional data were then analyzed at anatomically corresponding locations in all participants, based on this cortical alignment procedure. Thus, rather than testing activity in voxels, this analysis tests activity at vertices on the reconstructed maps. This analysis approach significantly increases the overlap of cortical regions across participants (Fischl et al. 1999; Frost and Goebel 2012; Goebel et al. 2006) and allows analyzing fMRI data without spatial smoothing. The Neuroelf toolbox by J. Weber (accessible at http://neuroelf.net) was used to access preprocessed data in Matlab for statistical analyses and ROI analysis.

#### Statistical analysis.

Data were analyzed using vertex-wise GLM. A random-effects group analysis was performed to test effects across participants. Each type of event in the experiment was modeled with a boxcar function that was convolved with a γ function that modeled the hemodynamic response of the blood oxygenation level-dependent (BOLD) signal. Our report focuses on the planning phase between target/effector specification and movement execution. Predictors were created according to instructed effector (hand, foot, or eye) and target side (left or right) of the current and previous trial, resulting in 3 current effector × 2 current target side × 3 previous effector × 2 previous target side = 36 predictors for the planning phase. As an example, one predictor covered an instructed hand movement to a left target that followed an instructed eye movement to a right target. The 20 s of rest at the beginning of a block were modeled as a baseline. Furthermore, there were six predictors for stimulus presentation (one for each target location) and six predictors for movement (3 effectors × 2 sides). Because the first trial of a run did not have a predecessor, these trials were modeled with one separate predictor. Each run ended with 8 s of rest to be able to record BOLD effects of the last trial. This end of a run was modeled by an own predictor. Trials in which participants made errors were modeled separately, with one predictor for each effector for the planning phase and one predictor for erroneous movement. Finally, several predictors were added to reduce noise in the model. We modeled head translation and rotation, as determined from motion correction during preprocessing, as well as the derivatives of these time courses with a total of 12 predictors. Furthermore, the model included the average out-of-brain signal, the average signal of the brain's white matter, and the average signal of the cerebrospinal fluid (Verhagen et al. 2008).

For terminology, we named conditions according the scheme “previous effector-current effector”; thus, for example, with “eye-hand,” we refer to those hand reach planning phases that were preceded by an eye movement trial. RS was investigated in the planning phase and defined as a decrease in fMRI activation when a trial was preceded by a movement of the same effector compared with when it was preceded by a different effector. By design of our experiment, RS effects can therefore be defined in several ways. For example, RS for the hand could be defined as hand-hand < eye-hand or as hand-hand < foot-hand or as hand-hand < (eye-hand plus foot-hand)/2.

Because our previous study had suggested that hand and foot movement planning activates very similar regions, we defined RS for the hand as hand-hand < eye-hand, for the foot as foot-foot < eye-foot, and for the eyes as eye-eye < (hand-eye plus foot-eye)/2. These contrasts identify regions that differentiate between eyes and limbs. We then defined two additional RS contrasts that differentiated between hand and foot, that is, hand-hand < foot-hand and foot-foot < hand-foot.

Using this scheme, RS effects will not emerge when neurons of a given region are active for all three effectors: in this case, neurons will always be repeatedly active, and, accordingly, no difference will result when contrasting effector repeat with effector nonrepeat conditions. Accordingly, lack of RS effects in a region that is active for all three tasks indicates that this region is task relevant but does not differentiate between effectors.

We used two approaches to analyze statistical significance. First, we assessed a cluster threshold for activation maps. This method computes the size that a cluster of activated vertices must have to be considered larger than expected by chance (Forman et al. 1995). Unless noted otherwise, the figures display activation maps thresholded at *P* = 0.05 (uncorrected) but indicate which regions remain significant after applying the cluster threshold procedure by outlining them with bold colored borders. Second, we selected regions of interest (ROI) on the cortical surface and assessed statistical effects in their averaged signal time course (see below). This approach allows testing for effects in regions for which prior hypotheses exist, even when activation of single voxels/vertices in these regions is not sufficient to survive corrections for multiple testing, albeit at the cost of reduced spatial resolution and brain coverage.

#### Definition of the ROI.

**Fig. 1.**
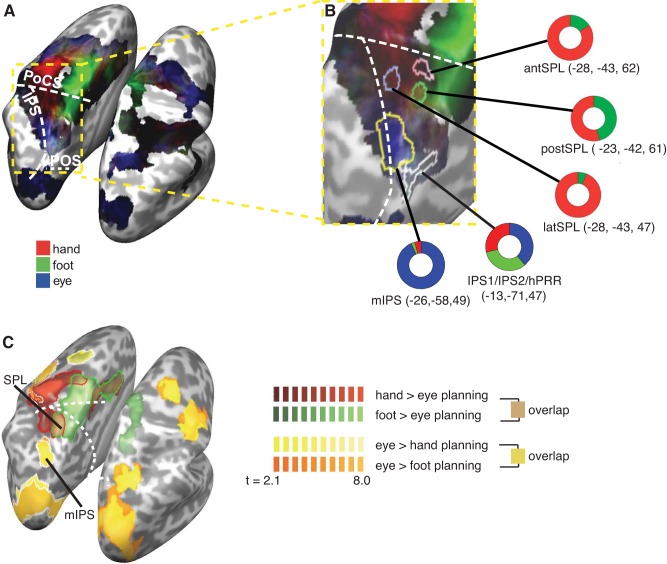
Frontal and parietal blood oxygenation level-dependent (BOLD) activation during motor planning for eyes, right hand, and right foot: gradient maps. *A*: comparison of the relative contribution of the β weights of the eyes (blue), hand (red), and foot (green) to explaining the BOLD signal during the planning phase. The brightness of the colors indicates the overall sum of the β weights, with brighter coloring indicating higher β weights (that is, “stronger” activation). The white dashed lines indicate the sulci. PoCS, postcentral sulcus; IPS, intraparietal sulcus; POS, parietooccipital sulcus. *B*: enlarged view of the area indicated by the yellow outline in *A*. Colored outlines demarcate the regions of interest (ROIs) we analyzed in the present study (see methods and Fig. 2, *A* and *C*, for their definitions). The colored rings visualize the relative size of the β weight of each effector in the respective ROI. antSPL, anterior superior parietal lobule (SPL); postSPL, posterior SPL; latSPL, lateral SPL; hPRR, homologue of the macaque parietal reach region in humans; mIPS, middle part of the IPS. *C*: activation maps for eye, hand, and foot pointing. Maps show the contrasts hand > eye planning (red), foot > eye planning (green), eye > hand planning (yellow), and eye > foot planning (orange). Maps are thresholded using a cluster threshold, with strong colored borders (red, green, and white) indicating regions that remain significant in the left hemisphere. Because the contrasts of eye > hand/foot planning are virtually identical, the result of the eye > foot planning contrast (orange) are largely obscured by the eye > hand planning contrast (yellow). Sulci are as in *A*.

We focused on five regions relevant to effector specificity. The coordinates we report here (see Fig. 1) are at the center of the ROI on our reconstructed surface.

The first ROI, comprising parts of the IPS1, IPS2, and hPRR, is referred to here as pIPS for brevity. It has been suggested that the cortex along the IPS is organized in adjacent, visuotopographically organized maps, termed IPS0-IPS5 (Konen and Kastner 2008). Activation for reaching and pointing has been consistently reported, among others, in two posterior regions, the IPS1 and IPS2, as well as medially of these, in a region which does not appear to be visuotopographically organized and has been suggested to be a homologue of the macaque parietal reach region (PRR), accordingly termed hPRR in humans (Connolly et al. 2003; Heed et al. 2011; Konen et al. 2013). The distances between the mean coordinates reported for the IPS1, IPS2, and hPRR are in the order of 1 cm (e.g., Konen and Kastner 2008; Schluppeck et al. 2005; Silver et al. 2005). In addition, these regions show considerable interindividual differences in location and extent (Konen et al. 2013). Here, we defined as pIPS the continuous reaching-related region in which contrasts for hand, foot, and eye planning (each against the rest baseline, that is, independent of the RS effects to be tested within the ROI) that was near or overlapped with the coordinates reported for the IPS1, IPS2, and hPRR.

The second ROI was functionally defined as the region more active during eye than hand planning. This ROI definition was independent of the RS effects to be tested within the region. The region was located in the middle part of IPS and termed here mIPS accordingly. The ROI definition was virtually identical when contrasting eye against foot rather than hand planning. We chose this region because evidence from multiple studies suggests that the IPS contains a region biased toward saccade processing (e.g., Hinkley et al. 2009), and RS effects related to saccade topography have been demonstrated here (Van Pelt et al. 2010).

**Fig. 2.**
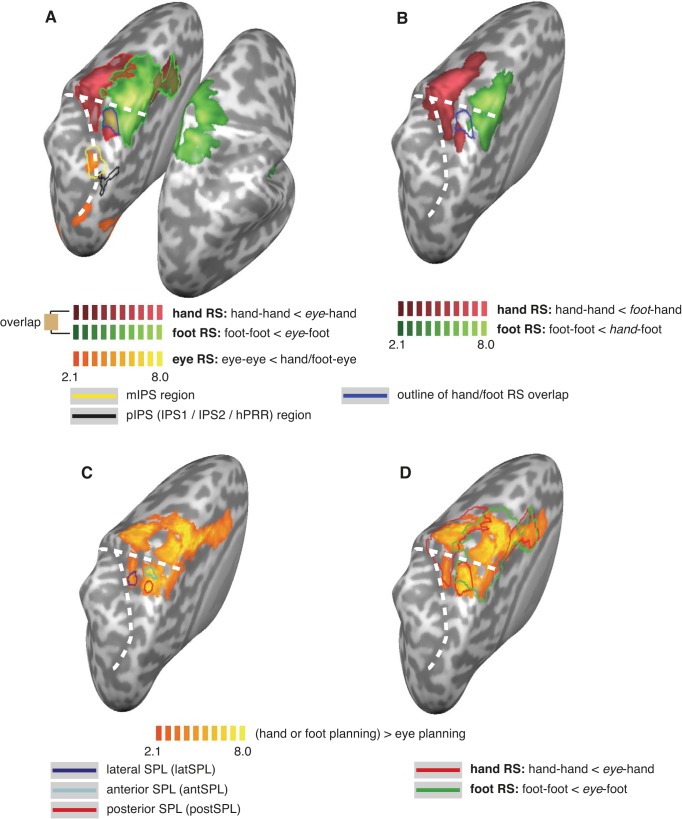
Activation maps for repetition suppression analysis. *A*: repetition suppression (RS) effects between the eyes and limbs, displayed as activation maps of contrasts hand-hand < eye-hand (red), foot-foot < eye-foot (green), and eye-eye < (hand or foot)-eye (orange). Bold colored borders indicate regions that survived cluster thresholding. The yellow outline indicates the region activated by eye > hand planning (see Fig. 1*C*). The black outline indicates the region near posterior parietal cortex (PPC) regions IPS1 and IPS2 that was activated by planning for all three effectors (see Fig. 1*B*). Brown coloring with dark blue border indicates hand and foot RS overlap. *B*: RS effects between the hand and foot, displayed as activation maps of contrasts hand-hand < foot-hand (red) and foot-foot < hand-foot (green). The cyan outline reproduces the eye-related hand and foot RS overlap from *A*. *C*: definition of ROIs. Activation for hand and foot versus eye planning is shown in yellow. ROIs were defined around the peaks of this contrast: latSPL (blue), antSPL (dark blue), and postSPL (red). *D*: same contrast as in *C*, but overlaid with the outline of the RS contrasts from *A*. Note that the ROI defined in *C* spatially coincide with the hand RS regions and hand/foot RS overlap region, although they are defined independently.

The remaining three ROI focused on contrasting limb and eye planning, based on our previous results that suggested similar coding for the limbs throughout the PPC. We contrasted the planning for hand and foot with eye planning (that is, disregarding RS, so that any RS analyses within regions selected using this contrast will be orthogonal). Because this contrast collapses across hand and foot, it will show activation when the planning of movements with either the hand, foot, or both drives the respective region relative to eye planning. The contrast revealed three peaks in the anterior SPL [Brodmann area (BA) 5 and BA 7]. We drew approximately circular, nonoverlapping ROIs around these peaks, including the most active voxels (see Fig. 2*C*). We refer to these ROIs as the lateral SPL (latSPL), anterior SPL (antSPL), and posterior SPL (postSPL) regions for brevity.

#### ROI statistics for RS effects.

For statistical analysis, the time course of all vertices (that is, the two-dimensional equivalent of voxels on the reconstructed cortical surface) within an ROI was first averaged. The GLM was then fitted to the averaged time course, resulting in a single β weight per predictor per subject. In each ROI, we then calculated five contrasts: RS for the hand (as hand-hand < eye-hand), RS for the foot (as foot-foot < eye-foot), RS for the eye (as eye-eye < foot-eye or hand-eye), and RS between the hand and foot (as hand-hand < foot-hand, and foot-foot < hand-foot). To account for multiple tests, we used the Bonferroni correction and report adjusted *P* values, that is, the *P* value resulting from the individual *t*-test multiplied by the number of tests conducted for the ROI.

#### Gradient analysis.

To visualize effector biases for all three effectors (see Fig. 1), we used the β weight for the planning predictors of each effector, weighted by the sum of all planning predictors, as color values (see also Heed et al. 2011). Biases for the eye, hand, and foot are displayed as blue, red, and green, respectively. In addition, brightness was used to express the overall activation of a voxel, with bright colors indicating high activation. This analysis was masked by contrasts of movement planning and execution.

#### Psychophysiological interactions.

Functional connectivity was investigated using the psychophysiological interaction (PPI) approach (Friston et al. 1997; Gitelman et al. 2003) based on the implementation in the SPM 8 package (available at http://www.fil.ion.ucl.ac.uk/spm/). We explored connectivity for two ROIs, the pIPS and SPL hand/foot overlap region, separately for hand and foot trials. Thus, four PPIs (2 effectors × 2 ROIs) were run. Accordingly, the psychological regressor comprised the planning regressors of the respective limb contrasted against the planning phases of the other limb as well as the eyes. Both movement trials and error trials were modeled as neutral (that is, the regressor was coded as 0). The physiological regressor contained the mean first eigenvalue of the ROI, corrected for all nuisance variables by regression. The psychophysiological regressor was deconvolved and multiplied with the psychological regressor to obtain the PPI regressor. Both the psychological and psychophysiological regressors were then convolved with a hemodynamic response function (HRF). The three regressors (HRF-convolved psychological, physiological, and HRF-convolved psychophysiological) were then used as predictors in a GLM, along with all nuisance regressors also used in our standard fMRI analyses. Statistical significance was assessed using a whole brain family-wise error-corrected cluster level threshold, based on a cluster-forming intensity threshold of *P* < 0.05 uncorrected.

## RESULTS

Sixteen participants performed delayed goal-directed pointing movements to visually defined target locations. In each trial, the instructed effector (hand, foot, or eye) was either identical or different to that of the previous trial.

### Behavioral Measures

Reaction time was comparable across effectors as well as for repeat versus nonrepeat trials [eye repeat: 507 ms (SE: 24 ms), eye nonrepeat: 515 ms (SE: 25 ms), hand repeat: 541 ms (SE: 30 ms), hand nonrepeat: 521 ms (SE: 28 ms), foot repeat: 551 ms (SE: 34 ms), foot nonrepeat: 548 ms (SE: 34 ms); repeated-measurement ANOVA with factors of effector and repetition, effector: *F*_2,30_ = 1.78, *P* = 0.20, repetition: *F*_1,15_ = 1.28, *P* = 0.28, interaction: *F*_2,30_ = 2.33, *P* = 0.11, Greenhouse-Geisser corrected].

More saccade errors, that is, saccades toward target stimuli and saccades accompanying instructed limb movements, were made after saccade trials than after limb trials [5.8% vs. 4.1%, χ^2^(4, 5) = 8.35, *P* = 0.004].

We assessed the correlation between target eccentricity and movement amplitude for each effector to ascertain that participants adjusted their movement amplitude to the target location in individual trials. The correlation between saccade amplitude and target eccentricity, computed separately for each participant and target side, was, on average, 0.56 (confidence interval of two SEs: 0.51–0.71; asymmetric interval is due to Fisher *Z*-transformation of correlation values for averaging). Similarly, both hand movement amplitude (0.60, confidence interval: 0.54–0.70) and foot movement amplitude (0.55, confidence interval: 0.50–0.67) were strongly correlated with target eccentricity. An analysis of Fisher *Z*-transformed correlation values with repeated-measurement ANOVA with factors of effector and target side did not reveal any significant differences for these factors (all *P* > 0.21).

### fMRI

#### Effects of motor planning.

We first analyzed motor planning in the delay phase for the three effectors. To this end, we averaged across repeat and nonrepeat trials, effectively making this analysis approach identical to that of our previous report (Heed et al. 2011) and allowing comparison of the two nonoverlapping participant samples.

Figure 1, *A* and *B*, shows that there was overlap for the planning of all three effectors along the IPS and dorsal PPC and a posterior-to-anterior gradient for eye versus limb activation. A gradient map weighting eye, hand, and foot activation in the planning phase showed largely overlapping activation along the left IPS and SPL, including a region near the superior parietooccipital cortex, in the region of visuotopographically defined areas IPS1 and IPS2. The overlap extended toward a region medially of IPS1/2, which has been suggested to be functionally homologous to the macaque parietal region (PRR) and termed hPRR (Connolly et al. 2003; Hinkley et al. 2009; Konen et al. 2013). Starting at the postcentral sulcus and extending forward to, and including, the premotor cortex, there was a strong bias for the hand on the lateral surface and for the foot on the medial surface of the left hemisphere, consistent with the known homuncular organization of the M1/S1 region along the central sulcus. In the sulcus of the medial IPS, there was a bias for eye movement planning, consistent with our previous results. Activation for all three effectors overlapped along all of the premotor cortex, with hints of somatotopy in the supplementary motor cortex. Contrasting the planning for each limb (hand, foot) with the eye (that is, hand planning > eye planning and foot planning > eye planning as well as the opposite contrasts) revealed a virtually identical activation pattern in the PPC (Fig. 1*C*). Hand- and foot-specific activation was evident only in the most anterior part of the parietal cortex. Eye-specific activation, in contrast, covered parts of the occipital cortex and a region in the medial part of the IPS. It is noteworthy that the contrasts of hand > eye and foot > eye overlapped anteriorly in the parietal cortex, in the bilateral SPL. Thus, this region, in both hemispheres, preferred the limbs over the eyes but did not show specificity for either limb in this analysis. Limb versus eye contrasts did not show activation in the dorsal PPC or in the region of the IPS1/IPS2.

Both the results from the gradient analysis as well as those from the contrast analysis were in close agreement with those from our previous report (Heed et al. 2011), thus replicating these findings with an independent participant sample.

#### Repetition suppression effects of eyes versus limbs.

Next, we analyzed fMRI RS effects as the difference between repeat and nonrepeat trials for each effector. Regions in which neurons are tuned to respond to a specific effector should show reduced hemodynamic activation for repeat compared with nonrepeat trials. This analysis is orthogonal to the prior analysis, because the conditions contrasted here were previously pooled together.

First, we tested for RS effects between eyes and limbs (Fig. 2*A*). To this end, RS for eye planning was defined as eye-eye < hand/foot-eye, RS for hand planning was defined as hand-hand < eye-hand, and RS for foot planning was defined as foot-foot < eye-foot. For eye planning, two left occipital/parietal regions showed RS effects at an uncorrected (*P* = 0.05) level but did not pass cluster thresholding. The first region was located along the very caudal end of the left IPS, just above the transverse occipital sulcus, within BA 19 (center Talairach coordinate: −28, −77, 19). The second region was in the left mIPS (coordinate: −24, −56, 44; Fig. 2*A*). The latter region overlapped with the activation resulting from the contrasts eye > hand/foot planning (see Fig. 1*C* and the yellow outline in Fig. 2A), in which activation for movement planning had been biased toward the eyes (see Fig. 1*B*, mIPS).

For hand planning, RS effects were evident in a large swath centered around the left M1/S1 hand region, extending anteriorly into the premotor cortex and supplementary motor area and posteriorly into the postcentral sulcus as well as onto the SPL (Fig. 2, *A* and *C*), both in BA 5 (i.e, von Economo's area PE) and anterior BA 7.

For foot planning, the anterior-posterior extension of RS effects was similar as that for the hand but was located medially, centered on the M1/S1 foot region (Fig. 2*A*). In addition, foot RS effects were evident bilaterally. Notably, RS was not evident in the center of the right hemispheric (ipsilateral) activation. For a contrast of foot movement against baseline, this region was the most active region in the left central sulcus (not shown). Therefore, we presume that the spared region in the right hemisphere is the M1/S1 region for the left foot (note that the homologous region in the left hemisphere is the most strongly activated region also for foot RS).

Importantly, there was a circumscribed left hemispheric (that is, contralateral) parietal region in which hand and foot RS effects overlapped, located in the SPL (coordinate: −27, −44, 62; see the brown patch with cyan outline in Fig. 2*A*). Interestingly, whereas the limb > eye contrasts had revealed hand and foot overlap also in the homologous SPL region of the right (that is, ipsilateral) hemisphere (see Fig. 1*C*), ipsilateral RS was evident only for foot planning, and, accordingly, overlap of RS effects for hand and foot was restricted to the contralateral hemisphere.

#### Repetition suppression effects of hand versus foot.

So far, we have defined RS effects for the limbs relative to the eye, that is, as hand-hand < eye-hand and foot-foot < eye-foot. In this scheme, overlap of hand and foot RS effects may indicate two different types of organization: on the one side, it could indicate that a region contains neurons that are active for both hand and foot but not eye planning. On the other side, the same result would be obtained if the overlap region contained an intermingled set of neurons sensitive exclusively to the hand and neurons sensitive exclusively to the foot. To differentiate between these two possibilities, we analyzed RS effects between the two limbs by defining RS for hand planning as hand-hand < foot-hand and for foot planning as foot-foot < hand-foot (Fig. 2*B*).

Limb-specific RS effects were evident around the left M1/S1 regions of the hand and foot, respectively, extending anteriorly into the premotor cortex and posteriorly into the postcentral sulcus. For the foot, this activation extended onto the right hemisphere. In contrast to the RS effects relative to the eyes, RS effects for hand and foot relative to each other did not overlap anywhere in the cortex. This suggests that there was no region in which hand-specific and foot-specific neurons were interspersed.

In Fig. 2*B*, the cyan outline illustrates the overlap of RS effects relative to the eyes (same as the cyan outline in Fig. 2*A*). Critically, only small patches of the overlap region with RS defined against the eyes showed limb-specific RS effects, suggesting that the SPL overlap region contained mainly neurons that respond to planning of both hand and foot and not to one specific effector. A region just posterior to the hand/foot RS overlap region was specific to the hand (Fig. 2*B*, red patch underneath the cyan outline), as it was activated in both the hand-hand < eye-hand as well as in the hand-hand < foot-hand RS contrasts (coordinate: −24, −54, 62). Similarly, the cortex directly medial to the overlap region was foot specific, in that it was active in both the foot-foot < eye-foot as well as in the foot-foot < hand-foot RS contrasts (Fig. 2*B*, green patch). Thus, neighbored by both hand- and foot-specific regions, we observed a limb-unspecific region in the SPL.

#### RS in ROIs.

Statistically speaking, nonsignificance, as for example observed for the hand versus foot RS, does not allow concluding that an effect does not exist. We therefore scrutinized RS results with an ROI approach to maximize the statistical power to detect potential RS effects for each effector. ROI analysis aims at circumventing the loss of statistical sensitivity due to correction for multiple comparisons by averaging, per subject, the time course over all vertices of the ROI and fitting a single GLM to this averaged signal. We defined five ROIs based on contrasts of limb versus eye motor planning and assessed RS effects within each ROI (see methods for details about independent ROI selection and definition and Fig. 1*B* for the corresponding activation results).

##### pips.

**Fig. 3.**
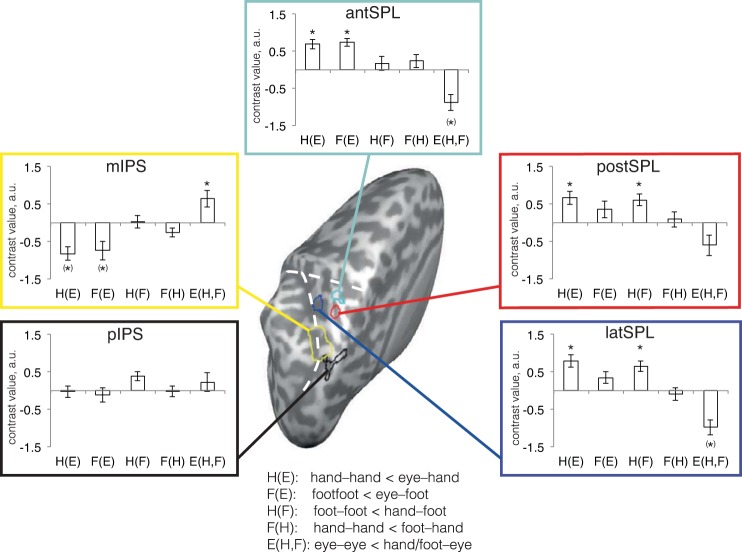
Contrast results for ROI analyses. The five analyzed ROI are illustrated on the reconstructed left hemisphere. Each ROI is accompanied by a panel framed with the same color as the ROI outline (coloring consistent with that in Figs. 1 and 2). Panels display the mean contrast value (contrast weights × β estimates) over participants for the five RS contrasts. E, eye; H, hand; F, foot. Thus, for example, H(E) indicates the contrast of hand RS relative to the eye, that is, hand-hand < eye-hand. RS is indicated by positive contrast values, that is, the difference of nonrepeat minus repeat trials. *Significance at *P* < 0.05, Bonferroni corrected for five comparisons. °Marginal effect at *P* < 0.10. ^(^*^)^Significance in the direction opposite to RS, that is, repetition enhancement. The white dashed lines indicate sulci (see Fig. 1 for details).

In the RS maps, no RS effects were observable in the region of the pIPS (the region spanning IPS1, IPS2, and hPRR), although activity was evident in this region for regular contrasts of planning versus baseline independent of the RS manipulation (see Fig. 1*C*). Analysis of an ROI covering this region confirmed this finding (Fig. 3, black box). None of the RS contrasts reached significance. It is important to keep in mind that RS effects for a given effector are expressed in relation to the other effectors. Thus, lack of RS effects does not indicate lack of activity. Rather, absence of RS will also be observed when neurons of a given region are similarly active for all tested effectors.

##### mips.

RS effects in the mIPS region, which had shown an eye planning bias (see Fig. 1, *A* and *C*), had not survived the cluster threshold. ROI analysis revealed a marginal RS effect for eye planning (*P* = 0.053, Fig. 3, yellow box; *P* value is Bonferroni corrected for the five tests devised in the ROI). None of the other contrasts were significant.

##### antspl.

**Fig. 4.**
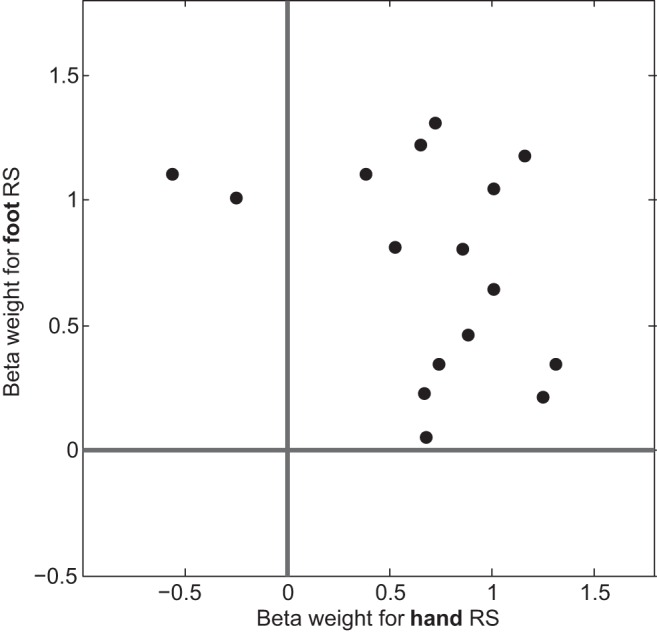
RS effects for single participants in the antSPL ROI, in which hand and foot RS overlapped. Each data point represents one participant's contrast values of hand RS (hand-hand < eye-hand) on the abscissa versus foot RS (foot-foot < eye-foot) on the ordinate axis. Zero axes are indicated by the dark strong lines. For the majority of participants, both hand and foot RS contrast values were nonzero.

A contrast of limbs > eye planning had revealed three peaks in the SPL. It is of note that all three regions spatially coincided with the regions identified using whole brain RS contrasts (cf. Fig. 2, *A* and *C*). The most anterior region, antSPL (Fig. 2*C*, cyan outline), largely coincided with the overlap region of hand and foot RS identified with the whole brain analyses (Fig. 2*A*). In this region, we observed hand and foot RS effects relative to the eyes (*P* < 0.001 for both limbs). More importantly, there were no significant RS effects between the two limbs (hand relative to foot planning, *P* ≈ 1 and foot relative to hand planning, *P* = 0.98; see Fig. 3, cyan box). We further tested the common activation for hand and foot RS by comparing activation for RS for the two limbs (each relative to the eyes) in each individual participant to ascertain that the overlap observed in the SPL was not due to two subsets of participants, one showing strong hand specificity and the other showing strong foot specificity, potentially averaging in the group to appear as nonspecificity. The β weights for hand and foot RS in the antSPL were above zero for the majority of participants (Fig. 4), suggesting that neurons in this region are sensitive to the planning of both hand and foot.

##### postspl.

The postSPL region, located just posterior to the antSPL, largely coincided with the hand RS patch identified in the whole brain analyses (Fig. 2*B*). This region showed hand RS against the eye (*P* = 0.001) as well as against the foot (*P* = 0.001). In contrast, it did not show foot RS to eye (*P* = 0.22) or hand (*P* ≈ 1; Fig. 3, red box).

##### latspl.

Finally, the most lateral ROI, latSPL, covered part of the large, hand-specific activation from the whole brain analyses. Its RS pattern was similar to that of the postSPL, with significant hand RS against eye (*P* = 0.008) and foot (*P* = 0.005) but no RS for foot against eye (*P* = 0.65) or hand (*P* ≈ 1, Fig. 3, blue box). Thus, ROI analysis confirmed that both the postSPL and latSPL had hand-specific RS patterns.

In an additional analysis, we calculated a GLM in which we modeled RS effects not just for the movement planning phase but also for the stimulation and movement execution phases. In such an analysis, there are higher correlations between the predictors for the different trial phases than in our main GLM analysis. Nevertheless, RS effects were consistently present only in the planning phase but not in the other trial phases. This result suggests that the effects we report are genuine to the process of movement planning.

#### PPIs.

**Fig. 5.**
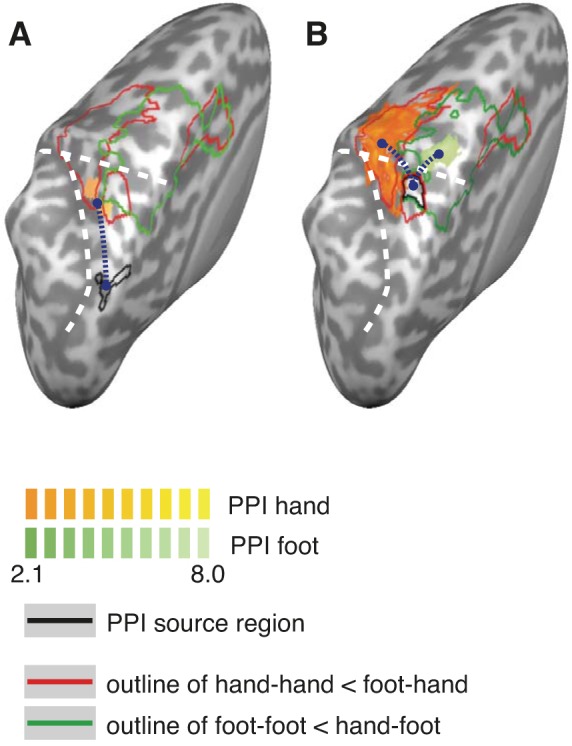
Psychophysiological interactions (PPIs). The dark blue dotted lines indicate presumed connectivity from seed regions (outlined in black in each panel) to the regions determined by the PPI analysis. *A*: PPIs for the pIPS region. The red and green outlines show the RS effects for hand and foot, respectively, relative to the eyes (see Fig. 2*A*). Functional connectivity during hand planning (orange patches) overlapped with the hand-specific RS region in the SPL but not with the hand-foot overlap region. A PPI for the foot did not reveal any significant activation. *B*: PPIs, separately for hand and foot trials, for the SPL RS hand-foot overlap region. Other details are as in *A*. For each effector (orange patches for the hand and green patches for the foot), functional connectivity from the SPL region was evident with the regions showing effector-specific RS effects. The white dashed lines indicate sulci (see Fig. 1 for details).

We wondered whether the regions showing RS effects during limb movement planning were functionally connected. We explored this possibility using, first, the pIPS region and, second, the SPL overlap region as seeds in a PPI analysis. For the pIPS, connectivity during hand movement planning was evident within a single region in the PPC. This region overlapped with the hand-specific RS region in the SPL (Fig. 5*A*). Connectivity was not observed with the SPL hand-foot overlap region. For foot movement planning, significant connectivity was not evident anywhere in the PPC for the pIPS. Thus, PPI analyses suggested a hand-specific connectivity pattern for the pIPS and did not support effector-specific connectivity between the two overlap regions in the posterior and anterior PPC.

For the SPL hand-foot overlap region (Fig. 5*B*), functional connectivity for hand movement planning was evident with the entire hand region that had been identified in our RS analyses, that is, lateral of the overlap region. For foot movement planning, connectivity was evident within the foot-specific RS region, that is, medial of the overlap region. Thus, PPI analyses suggested that information from the hand-foot overlap region in the SPL is selectively routed toward the motor cortex of the currently relevant limb.

### Alternative Account: Baseline Shifts

Inspection of Fig. 3 shows that there were repetition enhancement effects in some regions in addition to the RS effects we had hypothesized to observe (see negative RS effects for eye trials after hand and foot trials for the antSPL, postSPL, and latSPL). These reverse effects may indicate that what we interpret as RS effects are actually effects of a baseline shift. For instance, a region may respond more strongly to one effector, and a residual of this activation from the preceding trial might influence the effects of the next trial. We addressed this potential confound by reconstructing the BOLD signal time courses for each condition.

For this purpose, we constructed a model with nine regressors (3 current effectors × 3 effectors in the previous trial) and the same nuisance regressors as in the original model. The response was estimated with a finite impulse response function, modeling the full hemodynamic response for a given trial type using stick functions with an interval of 2.01 s (the TR). The prevalent finding was that the BOLD signal was affected by the previous trial's effector at the time of the current trial's stimulus presentation but then regrouped to reflect the current trial's effector. Most importantly, we did not observe long-lasting baseline differences that pertained through all trial phases and, thus, did not find evidence for this alternative account of our RS effects.

## DISCUSSION

Previous studies have suggested remarkably similar processing for the planning and execution of hand and foot movements in the PPC, calling into question the proposal that this region is organized in an effector-specific manner. We extended these studies using an fMRI RS approach, asking whether common fMRI activations (that is, spatially overlapping hemodynamic responses) during the planning of different effectors result from neurons genuinely responding to all effectors or, alternatively, from intermixed pools of neurons, each responding to only one specific effector.

We present four key findings. First, RS effects in the PPC followed a caudorostral gradient from eye planning (posteriorly) to limb planning (anteriorly), consistent with the previous proposition of an anterior-to-posterior, gradient-like organization of the PPC for eye versus limb planning (Burnod et al. 1999; Caminiti et al. 2010; Heed et al. 2011). Second, the IPS1, IPS2, and hPRR did not exhibit any RS effects, suggesting that these regions mediate planning for all tested effectors. Third, RS related to eye movement planning implicated regions previously associated with this effector, but were surprisingly weak. Fourth, the SPL displayed widespread RS effects that were mostly limb specific. However, there was a region that showed similar RS effects for the hand and foot and, accordingly, did not differentiate between the limbs. Information from this region appears to be routed to the effector-specific motor cortex depending on which limb is going to be used. Thus, fMRI RS analysis revealed both effector-specific and effector-unspecific PPC organization. We will first discuss each of these findings in turn and then place them in a larger framework.

### The Posterior-Medial PPC Does Not Differentiate Between Effectors

Planning for each of the three effectors activated a region along the PPC medial to the IPS and extending into the sulcus. This region overlapped with two areas defined by visuotopic mapping, IPS1 and IPS2 (Konen and Kastner 2008; Konen et al. 2013; Silver and Kastner 2009). No RS effects were detectable in the IPS1/IPS2/hPPR, here referred to as the pIPS, although this region was involved in the planning of all three tested effectors, as evident in significant activation against the rest baseline. This combination of results can be interpreted as indicating that the contributions of pIPS to motor planning are effector independent. Recall that RS was defined in the present study as the decrease of BOLD activation when a trial was preceded by an effector different from the current effector. Tentatively, as the interpretation is based on a null finding, the lack of any RS effects therefore suggests that, effectively, each trial repeated a feature coded by pIPS neurons, resulting in similar BOLD activation across all conditions. Alternatively, common BOLD responses for all effectors in this region may indicate that the region is related to other processes that are unrelated to movement planning, as for example to perceptual processing. We consider this alternative unlikely for three reasons. First, the pIPS region has frequently been associated with reach and saccade planning (Astafiev et al. 2003; Bernier and Grafton 2010; Filimon et al. 2009; Galati et al. 2011; Heed et al. 2011; Hinkley et al. 2009; Konen et al. 2013; Leoné et al. 2014; Prado et al. 2005; Vesia and Crawford 2012). Second, previous research has suggested that participants plan a motor response, rather than retaining sensory information, in delayed response paradigms such as the one used in the present study (Toni et al. 2002). Third, we separated stimulus, planning, and movement phases in our GLM analysis. Therefore, the effects we observed in the planning phase are unlikely to be related to stimulus processing.

### Weak Effector-Specific RS Effects for Eye Planning

A region within the middle part of IPS was biased toward eye movement planning (Hinkley et al. 2009; Schluppeck et al. 2005; Silver et al. 2005). Yet, although this activation spatially coincided with the putative human homologue of the macaque's eye-specific lateral intraparietal area (Grefkes and Fink 2005), eye-specific RS effects were weak and only marginally significant based on an independently defined ROI in the medial IPS. This may be surprising, given the amount of evidence for a bias, or even specificity, of this region for eye movement planning. In fact, RS effects for saccades have been demonstrated for presumably this same region (Van Pelt et al. 2010), based on the respective coordinates (reported coordinates −18, −59, 49 compared with those in the present study: −24, −56, 44). Crucially, Van Pelt and colleagues found RS effects only when saccades were repeated to the same location as in the previous trial, defined in eye-centered coordinates. In the present study, the stimulus location was never repeated because we intended to focus on effector specificity rather than spatial planning. Thus, the function of this intraparietal region may be strongly related to the definition of spatial targets in a retinal reference frame and only to a weaker extent to the eye as the relevant effector. Consistent with this interpretation, the region has been found to code not only upcoming saccades but also goal-directed hand movements in retinal coordinates (Medendorp et al. 2003, 2005).

### Anterior Parts of the SPL Contain Limb-Specific and Limb-Generic Regions

Hand and foot movement planning resulted in activation along the anterior wall of the postcentral sulcus and anterior SPL. These activations extended anteriorly across primary somatosensory and motor cortices up to the premotor cortex. We observed both limb-specific and limb-unspecific RS effects for hand and foot movement planning in the SPL. More lateral and posterior regions of the SPL were specific for the hand; the most medial regions were specific for the foot. The anterior SPL ROI, located between limb-specific regions and enclosed by the IPS and postcentral sulcus, showed RS effects for both limbs when compared against the eyes but not when compared against each other (that is, when RS was defined as hand-hand < foot-hand and foot-foot < hand-foot). These results imply that individual neurons in this region code for both limbs. The finding of common coding for the hand and foot in the SPL is consistent with several previous studies (Cunningham et al. 2013; Heed et al. 2011; Leoné et al. 2014). In contrast, the posterior SPL ROI and lateral SPL ROI showed RS effects of hand planning relative to eye and foot planning, implying hand specificity. Similarly, more medial and anterior regions showed RS effects for foot planning relative to both eye and hand planning, implying foot specificity. The different ROI were defined on the basis of specificity for limb compared with eye planning. In other words, limb RS effects were evident in regions that were selectively involved in limb planning, as identified independent of potential RS effects.

Because the hand and foot overlap region was wedged between hand- and foot-specific regions, the overlap we observed in our fMRI group analysis may actually reflect a mixture across subjects, with some subjects displaying hand specificity and others displaying foot specificity in the region identified by our contrasts. However, both hand and foot RS were evident within this region on a single subject level (see Fig. 4), discounting this possibility and affirming, instead, the conclusion that the neurons in this region truly code for both effectors.

The activation pattern of an effector-unspecific caudal PPC region (pIPS), an SPL region responsive to just two of the three investigated effectors, namely, the hand and foot, and effector-specific regions in the SPL for the hand and the foot, respectively, suggested that information may be routed between the caudal PPC and the hand/foot overlap SPL region as well as between this overlap region and the effector-specific motor cortex. In such a framework, information would gradually proceed from unspecific to increasingly more specific effector processing. Such information routing should be evident in selective functional connectivity patterns of the less specific to the more specific regions. However, we found only partial evidence for this hypothesis. We analyzed the connectivity patterns of the pIPS and anterior SPL. Connectivity was evident from the limb overlap region in the SPL to effector-specific motor regions for both hand and foot planning. In contrast, functional connectivity was not detectable from the pIPS region to the SPL limb overlap region. Therefore, it currently remains an open question which regions send information to the SPL overlap region to pass on further to the effector-specific cortex.

### Interpretational Issues

It might be argued that, in the present task, participants planned movements with all effectors concurrently, independent of the trial's instruction. Common activation patterns across conditions may then originate from parallel, effector-specific planning rather than from effector-unspecific representation. However, if this were the case, one would not expect any effector-specific activation patterns during the planning phase. Rather, activation differences should emerge only when the movement cue has been presented and the movement is executed. Because effector-specific activation patterns were evident in the anterior SPL for hand and foot and in the middle region of IPS for eye movement planning, it appears unlikely that common activation is due only to concurrent planning of movements with the different effectors.

RS paradigms are widely used in fMRI research (see, e.g., Grill-Spector et al. 2006), and RS paradigms have been used successfully for the investigation of motor planning (Bernier and Grafton 2010; Hamilton and Grafton 2009; Króliczak et al. 2008; Majdandžić et al. 2009; Monaco et al. 2011; Valyear et al. 2012; Van Pelt et al. 2010). RS effects have been previously reported in the regions studied here (e.g., Hamilton and Grafton 2009; Króliczak et al. 2008; Van Pelt et al. 2010). Still, the specific relationship between single neuron activity and fMRI BOLD activation is not well understood. Single neurons in the macaque's inferior temporal cortex showed higher neuronal RS for exact stimulus repetition than for presentation of a similarly effective, but different, stimulus (Sawamura et al. 2006). Importantly, RS was still observed in this latter case. In contrast, RS was not detected when a noneffective stimulus (that is, a stimulus that did not drive the neuron, independent of RS) was presented. The interpretation of these findings has been that RS, as observed in fMRI, is caused by interactions within the neuronal network of a region. Crucially, the presence of RS in fMRI is thought to indicate the presence of neuronal RS effects, albeit not necessarily in a proportional manner (Grill-Spector 2006; Hamilton and Grafton 2009; Sawamura et al. 2006).

Another caveat for interpretation is the absence of RS effects. Although RS effects have been demonstrated at comparably long lags as used in the present study (Hamilton and Grafton 2009; Króliczak et al. 2008; Van Pelt et al. 2010), they have been reported to be sensitive to interval timing (Henson et al. 2004). Therefore, the absence of RS effects in some regions in the present study does not preclude that such effects may be observed with differently timed paradigms.

Finally, some regions not only exhibited RS but also repetition enhancement (see Fig. 5). By means of an additional GLM analysis, we excluded that these reversed effects reflect baseline shifts induced by the previous trial. A related possibility is that RS effects were due to changes of the postmaximum dip of the BOLD signal. Because larger BOLD signal increases can lead to larger BOLD signal dips in the subsequent phase, it would seem possible that enhancement effects were caused by the same process as the RS effects. However, trials were maximally 8 s long in our experiment, whereas the dip of the BOLD response usually occurs 12–16 s after the eliciting event. Therefore, repetition enhancement in the present study is unlikely to be due to a dip of the BOLD response.

Although enhancement effects are not uncommon (e.g., Segaert et al. 2013), their interpretation is less clear than that of suppression effects. In the present study, enhancement effects were observed for the effector(s) complementary to those showing RS effects. The mIPS region showed RS for eye planning and enhancement for limb planning, whereas the antSPL and latSPL regions showed the reverse pattern. We did not observe such differences among hand and foot planning. Like in most studies that have compared eye and limb movements, participants in the present study had to suppress eye movements toward the target stimuli as well as during limb movements. Participants made more uninstructed eye movements after trials in which they had executed a saccade than after trials that required limb movements, suggesting that suppression of saccades was more difficult when an eye movement had recently been required. This difficulty effect may be related to the differences between limbs: for a limb RS trial pair, e.g., hand-hand, the first trial was sometimes, but the second trial was never, preceded by an eye movement. In contrast, for an eye RS trial pair, the first stimulus was sometimes, but the second stimulus always, preceded by a saccade trial, so that the effect of difficulty would be expected to be of opposite direction as that in limb trial pairs. Thus, the pattern of RS and enhancement effects in the antSPL, latSPL, and mIPS regions might indicate that these regions are involved in the suppression of saccades. However, given that the effect is in opposite directions in the SPL versus mIPS, one would have to posit that the SPL regions are more active, whereas the mIPS is less active, the more difficult saccade suppression. Moreover, this distinction does not account for the interpretation of the comparisons between the hand and foot and cannot explain the lack of differences of RS in the pIPS.

### Relation to Complementary Approaches

In a recent report, we investigated effector specificity in PPC in the subject sample analyzed here, but with an MVPA approach (Leoné et al. 2014). The present study investigated this issue on a yet smaller spatial scale, by looking at within-voxel neuronal sensitivity rather than across-voxel patterns. Previous studies have suggested that the two methods may possess different sensitivity (Sapountzis et al. 2010) or capture different aspects of neuronal processing (Drucker and Aguirre 2009; Epstein and Morgan 2012).

In the present case, MVPA and RS analyses revealed striking commonalities. Both methods are in agreement with respect to the posterior-to-anterior gradient of eye versus limb processing as well as to the effector-specific organization of the anterior SPL. Furthermore, MVPA identified a circumscribed anterior SPL region that differentiated between eyes and limbs but not between limbs, consistent with the present results for our anterior SPL ROI. MVPA also identified the mIPS to be dominant for the eyes, again consistent with the present RS findings. The MVPA and RS approaches revealed a difference only in the relative weighting of effectors in the anterior SPL. Using MVPA, we found equally strong representation of limbs and eyes, whereas the present analysis only detected RS for the limbs. Thus, RS appears to point more specifically to the limbs as the dominant representation, in line with the caudorostral eye-limb gradient.

The large-scale organization of the PPC has been investigated using several experimental approaches complementary to our present study: besides overt movement execution, a number of studies have either asked participants to imagine movements (often because their execution in an fMRI environment is difficult to control or, at the least, technically challenging to record) or to observe movies of another individual executing a movement. The present results relate to each of these approaches.

With respect to movement execution paradigms, activation evoked by visually guided bending of wrist and ankle overlapped in the SPL in a similar region as reported here (Cunningham et al. 2013), consistent with overlapping activation when participants sign their name with the hand and with the foot (Rijntjes et al. 1999). Furthermore, ataxic patients can show comparable deficits for both the hand and foot contralateral to the affected hemisphere (Evans et al. 2012; Rondot et al. 1977), although the often large size of lesions precludes a direct comparison with the present results. Single cell recordings and connection tracing in macaque monkey's area 5/area PE have revealed a homuncular organization of this region (Bakola et al. 2013; Taoka et al. 1998, 2000), although much less fine-grained than in the primary somatosensory cortex and with a strong overrepresentation of the hand (Seelke et al. 2011). Furthermore, the connectivity pattern of macaque area PE, with an emphasis on projections from regions involved in somatic sensation and from motor regions, together with a conspicuous lack of direct connections from visual areas, has been suggested to imply a role of this region in the coordination of movement in body-centered coordinates (Bakola et al. 2013). Although it has been emphasized that somatosensory area BA 2 and parietal area BA 5 have evolved in parallel with the emergence of skilled hand use in different species (Padberg et al. 2007), hand skills may ultimately have the same evolutionary basis as locomotion, that is, in humans, foot movement (Georgopoulos and Grillner 1989). This may explain the colocation of limb representations in BA 5. In addition, humans are quite skilled with their feet, as evident, for example, in dance, sports, and driving. Indeed, people born without arms can learn to use their feet for a wide range of tasks usually executed with the hand, like drawing and feeding.

With respect to imagery, choice of effector has been reported to be of less importance for fMRI activation patterns than task characteristics (Lorey et al. 2014). Furthermore, imagery of precision gait, compared with imagery of normal gait, revealed activation in the anterior SPL (Bakker et al. 2008). The authors of this study speculated that the function of their SPL region may be to predict the sensory consequences of planned movements.

Finally, action observation has been used to investigate the processing of more complex movements. For many activities, hand and foot movements must be precisely coordinated. The hand/foot overlap region we report here was activated in an observation study when participants observed locomotion but was activated even more strongly during observation of climbing, which requires coordination of hands and feet (Abdollahi et al. 2013). However, conclusions about the organization of motor-related functions based on experiments involving the observation of movement are not always straightforward. For example, observation of different motor actions, like pushing and pulling, activated regions in the inferior parietal lobule independent of the effector (mouth, hand, or foot) used to execute the action (Jastorff et al. 2010). These action-specific activations extended into regions previously thought to be hand specific, suggesting that the PPC is organized according to function rather than effectors. However, the motor-evoked potential induced by transcranial magnetic stimulation over the primary hand motor area was modulated when participants observed a foot performing an action typically executed by a hand (like grasping a pencil), suggesting that activations obtained in the context of action observation experiments may induce processes that are not genuine to purely motor-related tasks (Senna et al. 2014). Thus, it remains an open question whether or not the region we have identified to be active for both hand and foot planning is also involved in limb coordination. Answering this question will require experiments that test actual movement rather than action observation.

### Functional Role of the SPL

Viewed together, the diversity of these findings makes a simple organization of the PPC according to just one criterion improbable. In particular, these findings, along with the present results, do not support the idea that the PPC is organized according to the effectors involved in a movement. Instead, PPC organization appears to be largely effector unspecific in posterior regions, whereas some specificity may arise in the anterior PPC. Furthermore, MVPA of eye, hand, and foot planning activity has suggested that a central organizing principle of the PPC may be the selection of effectors by predominantly representing one effector as distinct from all others within PPC subregions (Leoné et al. 2014).

In this context, one can speculate about the role that the SPL hand-foot overlap region identified in the present study may play. The human SPL has been suggested to be crucially involved in the representation of the current postural state of the body (Pellijeff et al. 2006). A region whose neurons respond to all limbs would be perfectly fit for this function. Some studies that investigated posture reported activation in a region somewhat more posterior and medial than the overlap region we report (Pellijeff et al. 2006; Zimmermann et al. 2013). Another study involving blindfolded reaching to proprioceptively defined targets did report postcentral activation in a region that coincides with the present SPL overlap region (Parkinson et al. 2010) and suggested that it represented the body's postural configuration.

In monkeys, neurons responsive to specific, complex combinations of tactile and postural manipulations of several body parts have been found in macaque region PEc, located posteriorly to region PE (Breveglieri et al. 2008). However, whether or not this monkey region may be a homologue of the region identified here is currently purely speculative. Recently, single cell recordings were made in the dorsal part of macaque area 5 [dorsal and medial of the IPS, termed area 5d (Cui and Andersen 2011)] during a saccade-reach choice task. The location of area 5d appears to be slightly more anterior than PEc and may be comparable to the overlap region we report here. In fact, electrode placement was guided, among other parameters, by neurons' responsiveness to hand or foot stimulation. Neurons in area 5d encoded reaches (and not saccades) only after a choice for a reach had been made (Cui and Andersen 2011), and only the currently relevant reach in a sequence of two reaches (Li and Cui 2013).

In this context, the limb-specific activation in the SPL reported here may represent one stage of reach plan selection. Like monkey area 5d, the SPL region here shows activity for limb but not eye movement planning. Possibly, it represents the processing stage at which integration of the visual goal location is mapped to the body and from which information is then passed on to the effector-specific regions along the postcentral sulcus and premotor cortex. This account is compatible with RS effects in the SPL being unilateral in our study, given that the selected reach plan always concerns a right limb. Furthermore, it is compatible with the functional connectivity pattern obtained for this region, which was specific toward hand regions during hand movement planning and specific toward foot regions during foot movement trials.

### Conclusions

In conclusion, effector specificity is not an all-or-nothing concept. Mainly around the IPS1 and IPS2 regions, processing may be truly effector unspecific (but see Vesia et al. 2010). Thus, the functions mediated by these regions may be related mainly to target processing and be coded in a common reference frame independent of the potential effector used to respond. However, saccade processing differs in many regions across the PPC from the processing for limb movements, presumably due to the many differences inherent in both the function and execution of eye movements. These differences give rise to the posterior-to-anterior gradient of eye versus limb processing. Finally, for any type of limb movement, information may be routed through the limb-unspecific SPL to be relayed to specialized, limb-specific regions in the homuncular areas for final motor execution.

## GRANTS

This work was supported by the European Research Council (EU-ERC 283567 to W. P. Medendorp and EU-FP7-FET grant SpaceCog 600785 to W. P. Medendorp), the Netherlands Organisation for Scientific Research (NWO-VICI: 453-11-001 to W. P. Medendorp), and the German Research Foundation (DFG-HE 6368/1-1 to T. Heed). T. Heed is supported by the Emmy Noether program of the German Research Foundation.

## DISCLOSURES

No conflicts of interest, financial or otherwise, are declared by the author(s).

## AUTHOR CONTRIBUTIONS

T.H., F.T.M.L., I.T., and W.P.M. conception and design of research; T.H. and F.T.M.L. performed experiments; T.H. and F.T.M.L. analyzed data; T.H., F.T.M.L., I.T., and W.P.M. interpreted results of experiments; T.H. prepared figures; T.H. drafted manuscript; T.H., F.T.M.L., I.T., and W.P.M. edited and revised manuscript; T.H., F.T.M.L., I.T., and W.P.M. approved final version of manuscript.
